# An Italian pilot study of a psycho-social intervention to support family caregivers’ engagement in taking care of patients with complex care needs: the Engage-in-Caring project

**DOI:** 10.1186/s12913-019-4365-x

**Published:** 2019-08-02

**Authors:** E. Guida, S. Barello, A. Corsaro, M. C. Galizi, F. Giuffrida, G. Graffigna, G. Damiani

**Affiliations:** 1 Scientific Institute, IRCCS E. Medea 0-3 Center for the at-Risk infant, Bosisio Parini, Lecco, Italy; 2 Department of Psychology, Università Cattolica del Sacro Cuore di Milano, Milan, Italy; 3 EngageMinds HUB, Consumer & Health Engagement Research Center, Milan, Italy; 4 Health management Office, Social workers’ cooperative O.S.A, Rome, Italy; 5 Fondazione Policlinico Universitario A. Gemelli IRCCS, Rome, Italy; 6 Università Cattolica del Sacro Cuore, Rome, Italy

**Keywords:** Caregiver, Engagement, Engage-in-caring, Burden

## Abstract

**Background:**

The raising of disability and chronic illness burden among European population is calling for a new paradigm of care, focused on primary health care interventions. Engage-In-Caring is a novel multicomponent intervention clearly dedicated to improve family caregiver engagement in the care of patients with complex care needs, by supporting them to develop a stronger consciousness of their role, needs and skills.

**Method:**

Engage-In-Caring intervention’s efficacy and feasibility have been evaluated through a single arm pre-post observational pilot study settled in Rome. A qualitative phase, consisting of literature analysis of caregivers’ unmet needs and a final revision from an experts’ group, led to the structuration of the intervention, following the Caregiver Health Engagement Model (CHE-Model). Afterwards, a quantitative phase allowed understanding the feasibility of the intervention through Kruskal-Wallis test on a sample of 47 caregivers.

**Results:**

Results showed a reduction of the physical burden (Chi Squared = 6,483; *p* = .01) perceived by the caregivers and increase of the health literacy (Chi Squared = 3,560; *p* = .059) after the intervention.

**Conclusions:**

Feasibility tests on caregivers of patients with complex care needs are promising: this pilot study suggests a first effectiveness evidence, particularly concerning aspects related to burden perception and improvements in health literacy. Randomised controlled trials on larger samples are needed.

## Background

The raising of disability among European countries, together with the increased life expectancy at birth and the chronic illness burden - causing 86% of deaths worldwide -, are calling for a new paradigm of care that focuses on primary health care interventions and needs an active participation of patients and their caregivers [1–4]. For informal caregivers, this can lead to an increase in caregiver burden, due to the necessity of assuming more and more tasks for the person in need of care [5]. Informal caregivers are non-professional unpaid people providing care for a friend or a member of the family with a chronic illness, disability or other long lasting and complex health care needs [6–8]. They represent the primary form of chronic patients’ care in European countries providing 80% of the total burden of care, even in long term care performing countries [8]. Informal caregivers are mostly women, adult or elderly, spouses or mainly daughter, most of the time living together with the disable person; these conditions often lead to familiar overload with problems in reconciling familiar, parental and working activities and causing a big impact on the psychological and health status [9–12]. These physically and psychologically stressful conditions limit caregivers’ active engagement, their well-being and they can indirectly affect the patient who receive care [13]. Quite the opposite, it is well demonstrated that when a caregiver plays an active role in the team of care and is aware about his relevance, patients’ outcomes in both long and brief term are increased [14]. Nonetheless, to date there has been a lack of scientifically tested and structured interventions focused on supporting informal caregivers and embedded in the health care routine [15]. Literature suggests that helping caregivers to reach an appropriate level of health literacy, to be actively engaged and able to take care of their own wellbeing, is fundamental not to be overwhelmed and overloaded [13]. Nevertheless, actions on different components are needed to reach this condition: first, it is fundamental to support the family caregivers’ adjustment processes and their acceptance of the patients’ illness condition to enhance their ability to manage the psychological and physical burden [14]; moreover, family caregivers need a wider knowledge and a deeper literacy about the patient’s illness to become effective interlocutor of the healthcare system [14]; finally, communication and organizational skills are highly required [16]. Despite literature consensus regarding the need of actively involving caregivers in the management of their own relatives, there is a lack of interventions designed aimed at finding a sustainable balance between their caring tasks and their whole life projects, according to the multidimensional nature of this experience [17–19]. Indeed, caregiving research has focused primarily on caregiving associated with several specific patients’ populations (i.e., Alzheimer disease, cancer patients) and few studies have examined the effects of caring about chronic ill patients on caregivers themselves [20, 21].

This single arm pre-post observational pilot study presents an innovative and multifactorial intervention rooted in the Caregiver Health Engagement Model developed by Barello and colleagues and described in another publication [22]. The aim of the study is to better understand size and patterns of benefits deriving from an intervention specifically dedicated to caregivers of patients with complex care needs. Below the development stages of the Engage-in-Caring intervention and its feasibility test results are described.

## Methods

### Settings and participants

Study’s participants were main family caregivers, as well as those parents or friends more involved in the continuum of care of patients who were enjoying of OSA’s integrated home medical services. OSA “Operatori Sanitari Associati” is a Social Cooperative that handles home care programs on behalf of the Local Health Authority in Rome (Italy). The study lasted eleven months, from April 2017 to February 2018.

### Inclusion criteria

Subjects of both sexes aged more than 18 and fluently speaking and understanding Italian were  included in the study. Caregivers had to be the main familiar caregiver for at least one month and the patient doing at least 3 medications weekly (“intensive care” belonging Italian regulation on essential care) undertaken from the OSA’s integrated home medical service.

### Exclusion criteria

- To be in a psychological support training in the moment of the intervention’s proposal;
- Not to sign the informed consent;
- To have psychiatric pathologies;
- To be professional home nurses.

### Recruitment

The recruitment process consisted of three steps: identifying caregivers of patient with complex care needs patients; a first phase of telephonic contact; a second telephonic contact phase.

A first stage aimed at identifying principal caregivers of patients undertaken by OSA’s medical services. A “caregiver mapping study” has been realized in collaboration with the local health authority: among all the caregivers identified, only those who took care of patients receiving at least three medications per week have been enrolled in the study.

After this preliminary phase, we had a dataset including 267 contacts who were involved in the home care program. Nevertheless, among those 267 caregivers, it has been possible to contact only 138 people (129 people did not answer the phone, or the contact number was incorrect). Between those 138 people we were able to talk with, 91 refused to participate for different reasons: 35.2% lost the patient (death or hospitalization), 17.6% delegated to an in-home assistant, 12.1% refused without explanation and 11% refused because the patient did not agree (see Table 1 for more details).

**Table 1:** Non-adhesion reasons to the Engage-In-Caring intervention

Non adhesion reason	Frequencies	%
Refusal a priori/ no explanations	11	12,1
Delegation to in-home nurse	16	17,6
Perception to be Too old	3	3,3
Perception to be already engaged	5	5,5
Increased patient’s health	5	5,5
Non identified caregiver	4	4,4
Patent’s refusal	10	11,0
Multiple caregiver, strong network	5	5,5
Simple mortality (patient death, hospitalized)	32	35,2
Total	91	100

A third recruitment phase, which consisted of a new attempt of telephonic contact, allowed us to involve 5 more caregivers of critically ill patients.

### Ethics statement

Family caregivers were informed about the educational intervention procedures, and informed consent was obtained. The trial was approved by the local ethics committee (Prot. N. 478 CE ASL Roma 1, approved on the 2/3/2017).

### Assessment measures

We used self-reported questionnaires – described in details below - to explore different dimensions of the caregiving experience. Caregivers filled out every questionnaire just before the beginning of the intervention (T0) and one month after the end of Engage-In-Caring (T1).

Caregiver Burden Inventory (CBI). The Caregiver Burden Inventory is a multidimensional instrument useful to explore caregiver’s perception of burden. It returns five dimensions of burden: time-dependence burden, which evaluates caregiver’s stress related to the time the caregiver has to dedicate to the patient; developmental burden, that refers to hope and expectation of the caregiver; physical burden, that evaluates the physical stress of the caregiver, including health his own health and level of energy; social burden refers to conflicts in family and working context; emotional burden considers negative feelings the caregiver has against the patient. Every dimension goes from a minimum point of 0 to a maximum of 4. For every domain the global rate has a range of 0–20. The global rate goes from 0 to 100 [18].

Caregiving Health Engagement Scale (CHE-S). The Italian version of the CHE-s scale was used to evaluate the caregivers’ level of engagement in patients’ care management. The Caregiving Health Engagement Model features 4 main positions of engagement and shows that family caregivers who are engaged in the healthcare process are the ones who succeed in reframing their role and reaching balance between their caring tasks and their broad life goals. The CHE-s has been demonstrated to be a reliable measure to capture the fluctuating and dynamic nature of caregiver engagement resulting in a caregiver’s profile of engagement. Through an algorithm which provides the final score, it envisages four different positions along the engagement continuum – 1) denial, 2 hyper-activation, 3) drowning and 4) balance. The scale is measured on a 7-point scale in order to facilitate caregivers’ responses and to avoid social desirability bias [22].

Mishel Unertainity in Illness Scale (MUIS). The Mishel Uncertainty in Illness Scale (MUIS) has been developed in order to assess four aspects of uncertainty: ambiguity, complexity, inconsistency and unpredictability. It is a 5-item instrument with a good validity and has a high test-retest reliability. The scale is based on Mishel’s theory about uncertainty, which explains the importance of having a meaning for illness events, with uncertainty indicating the absence of meaning [23]. The items of MUIS are rated on 5-point Likert scale: 5 = “strongly agree”, 4 = “agree”, 3 = “I do not know— undecided”, 2 = “disagree”, 1 = “strongly disagree”.

Health Care Communication Questionnaire (HCCQ). The HCCQ is a questionnaire aimed to measure outpatients’ experience of communication with hospital personnel other than doctors. It is composed by 13 item and has a good factorial validity and scales reliability. The HCCQ gives information that could be taken as an indirect and subjective indicator of the quality of healthcare services [24]. Scores are calculated by averaging the individual item scores.

Health Literacy 3 item. The 3 item Health Literacy has been used to detect caregivers’ health literacy [25]. They were classified as having inadequate, marginal, or adequate. Responses were scored on a Likert scale from 0 to 4. The 3 items are “How often do you have someone (like a family member, friend, hospital/clinic worker or caregiver) help you read hospital materials?” (Help Read), “How often do you have problems learning about your medical condition because of difficulty understanding written information?” (Problems Reading), and “How confident are you filling out forms by yourself?” (Confident with Forms).

Revised scale for caregiver Self Efficacy. The Revised Scale for Caregiving Self-Efficacy measures 3 domains of caregiving self-efficacy: Obtaining Respite, Responding to Disruptive Patient Behaviors, and Controlling Upsetting Thoughts. Self-efficacy is a construct from Social Learning Theory and refers to a subjective belief that a person has about his or her ability to carry out successfully certain kinds of behavior. This measure contains 15 items within three subscales (self-efficacy for obtaining respite, responding to disruptive patient behaviors, and controlling upsetting thoughts about caregiving). Items are rated on a 0–100 scale [26].

### The qualitative phase: development of the “Engage-in-Caring intervention”

A preliminary literature analysis allowed us understanding and identifying principal caregivers’ unmet needs and questionnaires evaluating them, in order to structure the Engage-In-Caring handbook, a very easy to use tool containing also some suggestions and practical exercises.

**Fig. 1:**
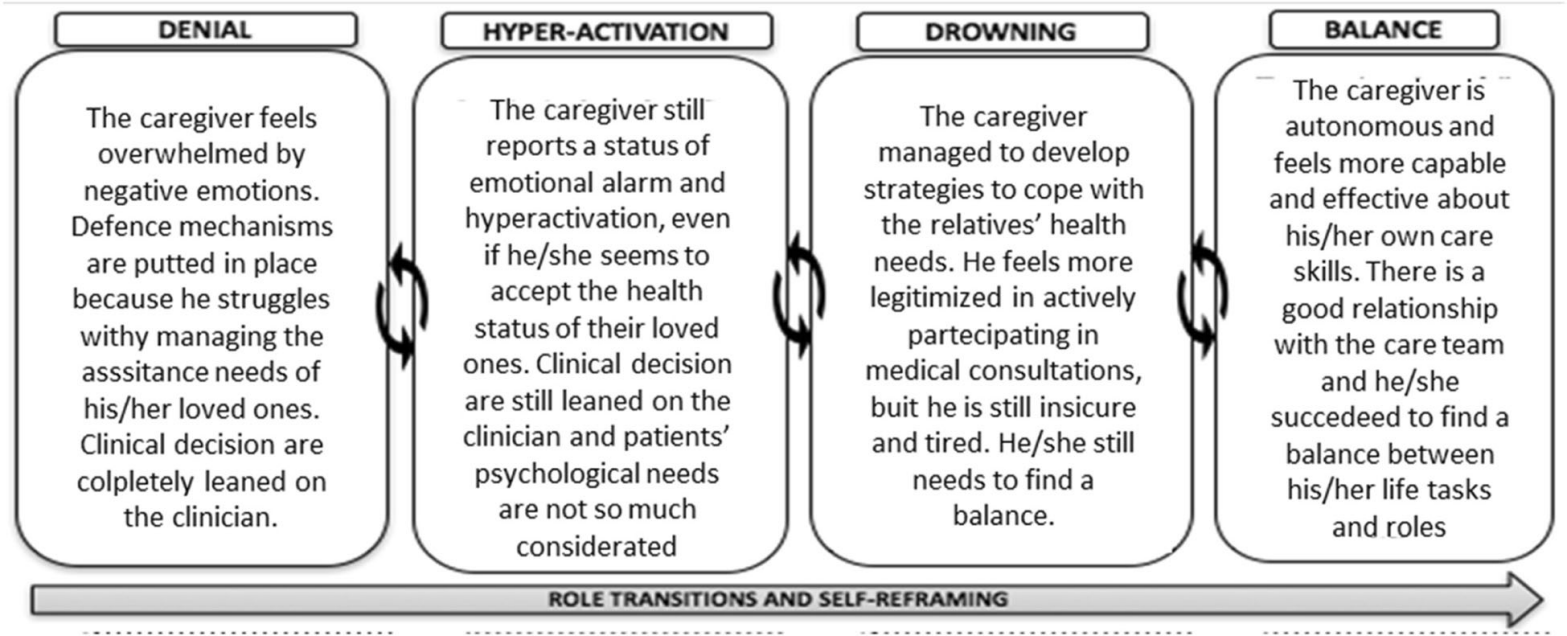
Description of the Caregiving Engagement journey

The theoretical framework that provides a structure in which to organize the Engage-In-Caring integrative intervention is the Caregiving Engagement Model [22]. This model describes the caregiving experience of taking care and its dynamical nature, consisting in four experiential positions: 1) denial, 2) hyper-activation, 3) drowning and 4) balance (Fig. 1).

Finally, an expert group of OSA health professionals analysed the intervention and gave their feedback and suggestions, leading the authors to develop a final version of the “Engage-in-Caring Intervention”.

### The quantitative phase: statistical analysis

Statistical Analysis have been run using IBM software for statistics SPSS 23. Our aim was to explore if any difference could be registered before and after the intervention concerning participants’ perception of their caregiving experience. After a descriptive analysis of the caregiver sample, we run the Kruskal-Wallis test in order to explore if there was any statistically significant difference between T0 (before the intervention) and T1 (after the intervention) for every questionnaire used in this study. ANCOVA has been run to test the possibility of a statistically significant influence of some socio-demographic characteristics.

## Results

### Qualitative results: the “Engage-in-Caring intervention”

The qualitative stages of this research gave life to “Engage-in-Caring”, an intervention developed to integrate the different areas crucial for the caregiver engagement experience. Indeed, literature analysis underlined how interventions to help caregivers are lacking. Particularly, Engage-in-Caring aimed to address the following objectives:

1. Information and communication management: to make caregivers effective in searching for and using healthcare-related information about their loved ones and in effectively interact with the healthcare team;
2. Learning to navigate healthcare organizations and getting organized: to make caregivers effective in navigating the healthcare organizations and managing their loved ones’ care requirements;
3. Taking care of their own wellbeing: to make caregivers able to take care of themselves;
4. Finding a balance: to make caregivers able to find a balance between their caring role and their other life tasks.

To obtain this, the intervention was thought to last overall one month and consisted of three motivational sessions and a self-administrated handbook.

The three motivational sessions provided by specialized nurses - who were previously trained about the aims and the contents of the program- were organized as follows: a) the first face-to-face session - lasting about 50 min - was planned at the beginning of the intervention path in order to present to the caregiver the aims of the intervention and its contents. In this meeting the nurse provided the caregivers with the handbook with a self-administrable handbook containing the educational contents/exercises to be used in the following month (see below for the more details about the handbook’s contents). Moreover, this meeting featured the administration of an evaluation survey to collect baseline data. b) The second phone-based motivational session – planned in the middle of the intervention period and lasting about 30 min - was aimed to collect caregivers’ ongoing feedbacks and to support their motivation to complete the intervention. c) The third motivational session was designed to collect caregivers’ experiences related to the intervention and to administer evaluation questionnaires.

The self-administrable handbook consists of exercises designed to address the four main objectives of the Engage-in-Caring intervention:

- To address the first objective - information and communication management - some exercises were developed in order to provide caregivers with tools useful to increase their awareness about knowledge and information related to their loved one’s disease and care requirements; moreover some exercises were developed to help caregivers in more effectively communicate and relate with the care team.
- To address the second objective - learning to navigate healthcare organizations and getting organized - some exercises were designed to help caregivers to effectively monitor their loved ones’ health parameters and life style habits.
- To address the third objective - taking care of own wellbeing - the handbook provides some tools to help caregivers in enhancing their awareness about the importance of taking care also of themselves and to support them in finding time and space to plan rewarding activities useful to reach a wellbeing status unless their caring role.
- To address the fourth objective - finding a balance - the handbook proposes narrative tasks to make caregivers able to psychologically elaborate their caring experience and tools to identify internal resources or external sources of support useful to make them able to find a balance between their caregiving role and their other life tasks.

### Quantitative results: statistical analysis

Table 2 shows basic descriptive data of caregivers’ sample, such as gender, age, sons and profession. Moreover, we explored who is the caregiver, if there is an in-home nurse and the duration of the caregiving period.

**Table 2:** Socio-demographic characteristics the caregiver sample (*N* = 47)

Patients’ Caregiver		Age	
Mother or Father	10%	30–50	35%
Sons	45%	51–60	17%
Wife/Husband	29%	61–70	26%
Sister/Brother	16%	71–80	13%
Years of caregiving		> 80	9%
1–5 years	22%	Job	
6–10 years	56%	Employee	24%
11–20 years	11%	Free lance	20%
21–34 years	11%	Retired	12%
Presence of in-home nurse	55%	Unemployed	4%
Sons (% yes)	69%	Housewife	36%
Gender		Other	4%
Female	83%		
Male	17%		

We tested if any statistically significant difference subsisted between T0, measured before starting the intervention, and T1, measured after the intervention.

Secondarily, we tested if the duration of the caregiving period and the presence of an in-home nurse affected the results running an ANCOVA. Results underlined that there was not any statistically significant influence of those elements on results shown below. To test the differences between T0 and T1, since the sample is not normally distributed, we decided to run a non-parametric ANOVA. Results of the Kruskal Wallis test highlighted some interesting aspects: the physical burden perceived by the caregivers, measured with the CBI, diminished significantly from T0 to T1 (Chi Squared = 6,483; *p* = .01); health literacy, instead, increased and it was very close to statistical significance (Chi Squared = 3,560; *p* = .059) (see Table 3). Moreover, there were some trends that can be underlined even if, probably due to the small sample, they are not statistically significant. For example, the caregiver engagement (CHE-S) increased (going from an average score of 1,36 to 1,57), as well as the quality of the communication with the clinician (HCCQ increased from an average score of 4,9 to 5,7) and the referred self-efficacy in managing disruptive behaviours of the patient (from 27 to 30); coherently with our expectations, the perceived uncertainty (MUIS) decreased (from a mean score of 15 to 14) similarly to the CBI perceived developmental burden (from a mean score of 13 to 11) (see Table 4).

**Table 3:** Kruskall-Wallis test results about Caregiver Burden Inventory: Physical Burden and Health Literacy (N = 47)

	CBI Physical Burden	Health Literacy
Chi Squared	6,483	3,560
Sig. Asint	,011	,059
T0 mean score	10,727	1,545455
T1 mean score	8,786	2,642857

**Table 4:** Mean scores at T0 and T1 of CHE-S, HCCQ, MUIS and Self-Efficacy in managing disruptive behaviours (N = 47)

	T0	T1
CHE-S	1,36	1,57
HCCQ	4,9	5,7
MUIS	15	14
Revised Scale for Caregiving Self-Efficacy: Managing Disruptive Behaviours	27	30
CBI: Perceived Developmental Burden	13	11

## Discussion

To our knowledge, Engage-in-Caring is the first multicomponent intervention clearly dedicated to improve engagement in caregivers, helping them to build a stronger consciousness of their role, needs and skills and consequently supporting them in becoming active players during the caregiving process and dynamics [15]. Those results represent the first feasibility test of the Engage-in-Caring intervention, showing clear improvements in caregivers’ health literacy and a diminished perception of their physical burden. Moreover, positive trends concerning their level of engagement, the quality of the communication with the clinician and the referred self-efficacy are interesting. Finally, the perceived uncertainty seems to decreases between the beginning and the end of the intervention.

Health literacy and burden perception are considered “main themes” for caregivers of complex care needs patients, because they are untrained and often feel not prepared to take on caregiving tasks. Indeed, they are the primary interface with the health care system, but often receive inadequate support from health professionals and frequently feel abandoned and un-recognized by the health care system [27]. For this reason, we consider of great importance the results of this pilot study, whose effectiveness evidence is mainly related to burden perception and improvements in health literacy.

A relevant aspect of this pilot study also regards the variety of users: Engage-In-Caring is not restricted to patients with a singular pathology, but embraces all caregivers who must face challenges of complex care needs patients. Preliminary results show positive trends on aspects such as caregiver engagement, quality of communication with clinician, self-efficacy in managing patient’s disruptive behaviours and perceived uncertainty. These study results are a first brick in showing the feasibility and effectiveness of an intervention aimed to help complex need patients’ caregivers. They fit in the varied panorama of psychosocial and pharmacological interventions aimed at mitigating caregiver burden and associated manifestations of caregiver distress. A meta-analyses review showed how both pharmacological and psychosocial existing interventions usually obtain mild to moderate efficacy; specifically, psychosocial interventions are characterized by support groups or psychoeducational interventions carried through counselling and individual trainings [28]. In this framework, Engage-In-Caring is an innovative intervention: it consists of a handbook containing some suggestions and exercise, so that it can be easily spread and used. Compared to other psychosocial intervention, this makes Engage-in-Caring a very easy to handle tool for its users; moreover, also the healthcare system can benefit from this kind of intervention, since the costs of an handbook are significantly inferior to face-to-face face trainings’ outgoing as well as less expensive than taking actions on the caregiver or patient uneasiness. For this reason, we suggest to involve caregivers in this kind of programme mostly when they are beginning their caregiving journey, when they are supposed to need more information on patients’ disease and management strategies especially in case of high intensity care patients [24].

### Limitations and future directions

Nonetheless, some limits need to be underlined: the small sample due to the low inclusion rate, the possible selection bias occurred because of the recruitment method and the lack of control group. The main focus of this study was to develop and pilot test an intervention structure for caregivers of patients with complex care needs and to and understand its feasibility. Future studies should evaluate the intervention effectiveness by using a RCT study design with larger inclusion rate. Moreover, future directions should assess not only the intervention’s effects on family caregivers but also its indirect effects on patients, for instance in terms of quality of life and clinical outcomes.

Finally, we had the intuition that when the suggestion for participation to the intervention comes from an operator they trust, is more likely that caregivers participate. This issue should be systematically controlled in future studies to determine whether this factor exists or not. Finally, we think that would be interesting to test a similar intervention on professional in-home nurses, that represent for 55% of families, other actors equally involved in patients’ care.

## Conclusions

Engage-in-Caring is an intervention recommended for caregivers who have to face challenges related providing support to patients with complex care needs in order to improve their engagement and promote their well-being, even if randomized control trials are needed to get stronger and more detailed evidences. Because of the potential highlighted by the results of this pilot study, we can state that the healthcare systems would benefit from the impact of a spread use of the Engage-In-Caring intervention. On one side, the quality of care would be enhanced thanks to the higher level of literacy and the ameliorated physical well-being of the caregiver: this more suitable condition of the caregiver may lead to a positive impact even on the patient, indirectly helping him to gain a higher quality of life despite the illness. On the other side, the caregiver itself will have a higher self-efficacy related to his role, consequently feeling more legitimate to take an active role with the clinician and the healthcare providers’ team. Higher health literacy, together with better communication skills and improved self-efficacy are fundamental elements to reduce uncertainty feelings and to help not only the patient, but also the caregiver to reach a status of balance and psychophysical wellbeing.

## Data Availability

The datasets used and analyzed during the current study are freely available to any scientist wishing to use them. The datasets used and/or analyzed during the current study are available from the corresponding author on reasonable request.

## Abbreviations

ANCOVA: Analysis of covariance
CBI: Caregiver Burden Inventory
CHE-Model: Caregiver Health Engagement Model
CHE-S: Caregiving Health Engagement Scale
HCCQ: Health Care Communication Questionnaire
MUIS: Mishel Uncertainity in Illness Scale
OSA: Operatori Sanitari Associati
